# Effect of Calcium Hydroxide and Chlorhexidine Medicaments on the Apical Seal

**Published:** 2012-03-01

**Authors:** Mahmoud Reza Hamidi, Elham Mahmoudi, Ali Akbar Moghadamnia, Samir Zahedpasha

**Affiliations:** 1. Department of Endodontics, Dental School, Babol University of Medical Sciences, Babol, Iran; 2. Department of Pharmacology, Medical School, Babol University of Medical Sciences, Babol, Iran

**Keywords:** Calcium Hydroxide, Chlorhexidine, Dental Leakage, Intracanal Medicament, Sealing

## Abstract

**Introduction:**

Leakage of the root canal system is an importent consideration when placing an intracanal medicament. The aim of this in vitro study was to compare the effect of calcium hydroxide and 1% chlorhexidine gel as intracanal medicaments on tooth apical seal.

**Materials And Methods:**

Seventy extracted, single-rooted maxillary anterior teeth were divided into the three experimental groups (n=20). All root canals were instrumented with step-back technique and divided into three groups. Group 1 had root canal dressing with calcium hydroxide; group 2 had root canal dressing with 1% chlorhexidine gel and group 3, did not receive a dressing. The roots were incubated in 100% humidity at 37°C for 7 days. After removing the dressings, all canals of the experimental groups were obturated using a cold lateral condensation technique. The root surfaces of all specimens were coated with two layers of nail varnish, except for the 2 mm surrounding the apical foramen. Apical sealing ability was assessed by dye leakage method and the specimens were examined under a stereo-microscope. Dye penetrations were measured and analyzed using ANOVA and post-hoc Tukey test.

**Results:**

Calcium hydroxide group had the least frequency of apical leakage at 2 mm level (0.46±0.40 mm), whilst chlorhexidine group showed the greatest apical leakage (0.86±0.42 mm). There was statistical difference between group 1 and 2 (P<0.05), but no statistical difference between group 1 and 3, or between groups 2 and 3 (P>0.05).

**Conclusion:**

Intracanal calcium hydroxide medicament may decrease apical leakage of gutta-percha root fillings when AH26 sealer is used; chlorhexidine may increase the leakage.

## Introduction

The aim of endodontic treatment is to remove bacteria and their by-products from the root canal system and to create a tight seal that prevents reinfection [1]. This seal is developed to minimize the leakage along root canal fillings and to protect the periapical tissues from bacteria and their by-products [2][3]. Coronal leakage is an event implicated in all stages of endodontic therapy, and it might lead to treatment failure [4]. Intracanal medications are used to eliminate bacteria in the root canal, prevent bacterial proliferation between appointments, and act as a physiochemical barrier, preventing root-canal reinfection and nutrient supply to the remaining bacteria [5]. The influence on leakage of the root canal system is an important issue when inserting an intracanal medication [6].

In modern endodontics, calcium hydroxide [CH; Ca(OH)_2_] is the most commonly used intracanal dressing [7][8]. It has been clinically used to obtain microbial control, dissolve organic remnants, heal periapical inflammation, inhibit inflammatory root resorption, stimulate hard tissue formation and serve as a temporary obturating material between appointments [7][9][10]. Its antimicrobial action is related to its high pH, which results in the inactivation of bacterial membrane enzymes [11][12][13]. However, CH has been found to be insufficient for elimination of both facultative anaerobes and yeasts [14][15][16]. It is believed that the removal of the CH paste before obturation of the root canal is important in obtaining a hermetic seal of the permanent root canal filling [7]. It has been suggested that CH remnants should be removed with further instrumentation of the medicated canal using files [17]. Porkaew et al. have reported that the residual CH along the root canal walls enhances the sealing quality of the root canal filling [18].

Chlorhexidine gluconate (CHX), a cationic bis-biguanide with optimal antimicrobial action over the pH range 5.5-7.0, has been suggested as an irrigation solution/intracanal medication [19][20] because of its strong antibacterial activity against gram-positive and gram-negative bacteria as well as yeast, facultative anaerobes and aerobes [21][22][23][24]. Chlorhexidine was introduced to increase the antibacterial effect of intracanal medications and to eliminate microorganisms associated with persistent infections and treatment failure [25]. In addition to its exceptional antimicrobial activity, another favorable property of CHX is substantivity [26][27]. Komorowski et al. demonstrated a 7 day substantivity of CHX in bovine incisors [26]. These characteristics have led to the possibility to use CHX as an intracanal medication.

To date, only few studies have investigated whether intracanal placement of 2% chlorhexidine gel affects on the sealing ability of an obturated root canal system [28][29].

The aim of the present in vitro study was to compare the short-term sealing abilities of permanent root canal fillings after the placement of CH and 1% chlorhexidine gel when used alone with a dye-leakage model.

## Materials and Methods

### Preparation of specimens

A total of seventy freshly extracted, single-rooted maxillary anterior teeth were used in this study. Calculus and soft tissue debris were removed with scalers. After tooth decoronating at the level of cemento-enamel junction, the working lengths were determined by placing a #10 file into the root canal until it was visible at the apical foramen and subtracting 1 mm from that length. Instrumentation of the root canals was serially done to a #40 K-file as master apical file (MAF). Step-back technique was used for the subsequent 4 larger sizes files (up to #60). The coronal one-third was flared with no 2, 3 and 4 Gates Glidden burs. The root canals were conventionally irrigated with 1 mL of 5.25% NaOCl after use of every other file. After the root canals received a final irrigation with 10 mL of 5.25% NaOCl and were dried with sterile paper point. The roots were randomly divided into three groups 1, 2, and 3 (n=20). An additional ten roots were used for negative and positive leakage controls. All the root canals were prepared by one operator.

In group 1, the root canals were filled with CH paste. Powder of CH (Henry Schein Co., Melville, NY, USA) was mixed with distilled water at a powder to liquid ratio of 6:4. The paste was introduced using #35 lentulo spiral filler (Antaeos, Munich, Germany) and packed with a plugger (Schilder plugger, 0.7 mm, Dentsply, Ballaigues, Switzerland) into the prepared canals until the CH was extruded beyond the apical foramen. Three millimeters of CH was then removed from the coronal part of each root canal.

In group 2, CHX gel 1% (Corsodyl Dental Gel, GlaxoSmithKline, Philadelphia, USA) was placed into the root canals with an Ultradent Capillary Tip (Ultradent Products Inc., South Jordan, Utah, USA). Gel was packed with the use of a cotton pellet until the CHX was visible at the apex.

In group 3, no medication was introduced. The access openings were sealed with a 1 mm cotton pellet and a 1 mm layer of IRM (Dentsply Caulk, Milford, DE, USA).

All the teeth were wrapped in water saturated 2×2-inch gauze, and stored at 37°C at 100% relative humidity for 7 days. At that time, the temporary material was removed with a slow-speed #2 round burs. After removing the dressings via irrigation with 5.25% NaOCl and by reaming with #40 K-file, all the root canals were obturated with gutta-percha and AH26 root canal sealer (Dentsply, Konstanz, Germany) by using the cold lateral compaction technique. The access cavity of each root was filled with a light-curing composite resin (Metafil CX, Sun Medical Co. Ltd., Moriyama, Japan). After the obturation, radiographs were taken to confirm the perfection of root obturations. Obturated roots were then stored in gauze and placed in an incubator for 72 hours at 37°C and 100% humidity sealer setting. The root surfaces of all specimens were coated with two layers of nail varnish, except for the area surrounding the apical foramen. Five teeth were covered completely with nail varnish following the obturation of the root canals, to serve as the negative controls. Another 5 teeth, which received no root canal fillings but had a varnish coating except at the apex, were used as positive controls.

### Dye-leakage test

Each group was immersed in India ink separately and kept at 37°C for 1 week. After thorough wash of teeth under running water and removing the nail varnish with a scalpel blade, the teeth were demineralized in 5% hydrochloric acid, washed in tap water for 24 hours, dehydrated in ascending concentrations of ethanol, and finally transferred to methyl salicylate for diaphanization. The cleared teeth were analyzed by means of a stereomicroscope (×40 mag.). The amount of apical leakage scored independently by three investigators through assessing the greatest extent of the dye.

### Statistical analysis

The data were analyzed using ANOVA and post-hoc Tukey test. The significance level was set at 0.05.

## Results

The linear measurements of dye leakage in the experimental groups are presented in Table 1 .

**Table 1:** Linear measurement of leakage in different experimental groups

	** Linear leakage (mm)**			
**Groups**	**N**	**Mean (SD)**	**Min**	**Max**
** 1^a^**	20	0.46 (0.40)	0.0	1.5
**2^b^**	20	0.86 (0.42)	0.43	2.07
**3^c^**	20	0.73 (0.24)	0.3	1.13

^a^ Calcium hydroxide-medicated group

^b^ Chlorhexidine-medicated group

^c^ Non-medicated group

The specimens of CH medicated had the least frequency of apical leakage at the 2 mm level (0.46±0.40 mm), whilst group 2 had the greatest (0.86±0.42 mm). Although, there was significant statistical difference between group 1 and 2 (P<0.05), there was no significant difference neither between groups 1 and 3, nor between groups 2 and 3.

The dye leakage of the negative controls was uniformly zero, whilst the positive control showed leakage of the dye all along the length of the root canal.

## Discussion

The sealing ability is considered to be very important for root canal filling materials. In the present study, CH medicated group showed the least frequency of apical leakage at the 2 mm level, whilst CHX medicated group had the greatest frequency of leakage. An improvement in the sealing quality of root canal fillings with different sealers has been reported when CH was used as a temporary dressing [18][30][31]. This was explained in the following two hypotheses: i) the residual CH is incorporated into the sealer during obturation, which may cause a decrease in the permeability of the sealer itself and ii) CH penetrates or is mechanically forced into the dentinal tubules, blocking them off and decreasing permeability [18]. Researchers reported that apical leakage was less in teeth that received CH dressings than non-medicated control teeth [18][30]. However, in a study by Kim and Kim, CH medicated root canals showed significantly more apical leakage than ones without medication [32]. Because CH cannot be removed completely from the canal, it is likely that the remaining CH may interfere with the seal ability of fillings when a zinc oxide-eugenol sealer is used. Chlorhexidine in the chemical form is a cationic bis-biguanide and has a wide spectrum of antibacterial activity [19][21][22]. It has been shown to be both bacteriostatic and bactericidal at low and high concentrations respectively. It has the ability to be adsorbed in the dentin and gradually released over time [19][27][33]. As an intracanal medication, CH was found to be as effective as 1% CHX in reducing Enterococcus (E.) faecalis at 3 and 8 days, whereas at 14 days, the efficiency was found to be less than the other test periods [34]. In comparing 0.2% CHX irrigation with 5% CHX slow-release device, the controlled release device was significantly better in eliminating E. faecalis growth at various dentinal depths [35]. 2% CHX produced the same inflammatory response as phosphate-buffered saline in mice [36]; the use of a 2% CHX as a periodontal irrigant did not cause obvious toxic effects on gingival tissue, suggesting its safety for intraoral use [37]. Therefore, these properties make 2% CHX gel a suitable alternative to CH as an intracanal medication. Wuerch et al. indicated that using 2% CHX gel as an intracanal medication for 2 weeks does not adversely affect the apical seal when using AH Plus sealer [29]. Kontakiotis et al. found that the new paste made of CH plus 2% CHX gel can be proposed for use in clinical practice without affecting the sealing ability of root canal obturation [28].

Wu et al. claimed that the results from dye leakage studies using the methylene blue were questionable, because the dye was found to be decoloured by CH [38]. The decolorizing effect of CH is related to its high alkalinity and this varies according to its form being as paste, cone or sealer [38][39][40][41]. In this study, India ink was used as a marker because it was not shown to be decolourized by CH [7]. Other advantages of using this dye are that it does not stain the dentine and shows the leakage pattern only. In addition, the extent of dye penetration is easy to detect [42] even in the dentinal tubules [43].

## Conclusion

Under the conditions of this in vitro study, applying 2% CHX medicament gel for one week adversely affects the apical seal of gutta percha and AH26 sealer. However, CH medicated canals showed the least apical dye-leakage. As this study used large and straight canals over a one week period, we recommend the evaluation of the long-term effects in teeth with small or curved canals.
